# Reasons for Using Roll-Your-Own Tobacco and Perceptions of Health-Promoting Pack Inserts: A Focus Group Study with Roll-Your-Own Tobacco Smokers in Scotland

**DOI:** 10.1093/ntr/ntac184

**Published:** 2022-07-30

**Authors:** Crawford Moodie, Rachel O’Donnell

**Affiliations:** Institute for Social Marketing and Health, Faculty of Health Sciences and Sport, University of Stirling, Stirlingshire, UK; Institute for Social Marketing and Health, Faculty of Health Sciences and Sport, University of Stirling, Stirlingshire, UK

## Abstract

**Introduction:**

Use of roll-your-own (RYO) tobacco is increasing in most regions, but few qualitative studies have explored why RYO smokers use this product, and no study has considered their views of health-promoting pack inserts.

**Methods:**

Eight focus groups were conducted with 18–35-year-old RYO smokers (*n* = 50) in Greater Glasgow (Scotland) in February–March 2020 to explore reasons for using RYO and perceptions of health-promoting inserts. Participants were shown four inserts adapted from those required in cigarette packs in Canada, with all encouraging quitting, and two RYO-specific inserts explaining that RYO is not less harmful than cigarettes.

**Results:**

Lower price, better taste, the pleasure of rolling and ability to customize roll-ups, and the belief that RYO was less harmful than cigarettes were drivers for use. There were mixed perceptions of the extent to which inserts would capture attention if included in RYO packs. The positive messaging used on the Canadian inserts was considered motivational and inspirational, and contrasted with the on-pack warnings. The messaging on the RYO inserts, in comparison, was viewed unfavorably and generally dismissed. Participants, most of whom were not interested in quitting, did not feel that inserts would lead them to change their smoking behavior. However, some felt that the Canadian inserts could be helpful for those thinking about quitting and young people contemplating smoking.

**Conclusions:**

Inserts with positive messaging about quitting, rather than messaging explicating the harms of RYO, were preferred by RYO smokers. What, if any, RYO-specific messaging resonates with RYO smokers merits further attention.

**Implications:**

Aside from price, taste, and the pleasure associated with rolling and ability to individualize roll-ups, the erroneous belief that RYO is less harmful than cigarettes was a key reason for use. While inserts with positive messaging about quitting, as used on the Canadian inserts, were viewed as potentially helpful, inserts that challenged the idea that RYO was not less harmful than cigarettes were generally dismissed. Research is needed to understand what types of RYO-specific messaging could most effectively be used on inserts, or indeed in other media, to challenge the misperceptions that many RYO smokers hold.

## Introduction

While roll-your-own (RYO) tobacco remains a niche product, globally, it is a growth category, with sales increasing in South America, Asia, Oceania, the Middle East and Africa.^1^ The highest demand is in Europe.^2^ RYO sales rose in Europe from 53,000 tonnes in 2000 to 82,000 tonnes in 2015,^3^ with the continent home to the nine largest RYO markets in the world by 2016.^4^ RYO is particularly popular in the United Kingdom, where smoking prevalence is 14.1%,^5^ being used by approximately two-fifths of adult smokers.^6,7^ RYO smokers are more likely to be younger, male, more addicted, from deprived areas, and less inclined to quit than cigarette smokers.^8^ Since 1990, not only are more smokers using RYO in the United Kingdom, but also exclusive use has increased.^9,10^ There is no sign of this trend changing given that RYO is the fastest-growing tobacco category in the United Kingdom, with a £684 million increase in sales in 2020^11^ and sales growth of 41.6% during the year to March 2021.^12^

A number of academic surveys have explored reasons for RYO use. Lower price is a key driver.^13–16^ RYO use is also associated with a stronger belief that it is less harmful than cigarettes,^13–18^ as well as better taste, greater satisfaction and a desire to reduce consumption.^13,14^ Qualitative research has additionally found that RYO smokers enjoyed the ritual and skill in rolling a cigarette and the therapeutic benefit of doing so, viewed RYO as more natural, and considered there to be social benefits as smoking roll-ups helped them make friends.^19,20^ Industry research also points to other reasons for use, with RYO permitting users to express their individuality by creating and personalizing their cigarette (eg, size, length, type of paper) and thus shaping their smoking experience.^21,22^ In this study, we explore reasons for RYO use in a market with standardized packaging, which was fully implemented for cigarettes and RYO in the United Kingdom in May 2017. Standardized packs have pictorial warnings covering 65% of the main display areas and two additional text warnings (“Smoking kills – quit now” and “Tobacco smoke contains more than 70 substances known to cause cancer”) on the sides of cigarette packs or inside of pouches.

In terms of potential avenues for discouraging RYO use, the packaging could be used to challenge the belief that RYO is less harmful than cigarettes,^23^ a view prevalent among RYO users.^13–18^ This information could be delivered via warnings on the outside of the pack or messages on inserts inside of the pack. We focus on pack inserts as there is growing policy interest in their potential value as a means of communicating with consumers. Inserts were introduced in Canada in 2000, although only required in packs of cigarettes and little cigars. Sixteen text-only inserts were required in packs until 2012, with nine encouraging cessation and seven providing health risk information.^24^ These were then replaced with eight new inserts, with colored graphics and positively framed messages about the benefits of quitting or tips on how to do so. Israel became the second country to legislate for inserts in 2018^25^; the inserts, currently being developed, will be required in all tobacco and vaping products.^25^ In the United Kingdom, inserts have been recommended as part of the Tobacco Control Plan 2030.^26^ There is, however, limited research on inserts,^27–35^ and no qualitative work exploring the views of RYO smokers.

Given the popularity of RYO in the United Kingdom and growth elsewhere we explored why RYO smokers use this product, and whether health promoting inserts may be perceived as having the potential to discourage use.

## Methods

### Design and Sample

We conducted eight focus groups segmented by gender and age (18–24, 25–35), with daily RYO smokers (*N* = 50) in Greater Glasgow in Scotland between February-March 2020. We explored reasons for using RYO and perceptions of inserts. Participants were recruited in Greater Glasgow, using street intercepts, by an experienced market researcher using convenience sampling. The market researcher explained that the study was concerned with perceptions of RYO tobacco and packaging. Demographic information (age, gender) and smoking behavior (smoking frequency, consumption) was captured by a recruitment questionnaire (see Table 1). The inclusion criteria were that participants smoked RYO every day, as we were most interested in regular users, and were within one of the gender and age groups.

**Table 1.** Characteristics of Participants in Each Focus Group

Group	Gender	Age range	Product preference	Daily RYO consumption(range)	Number of participants
1	Female	18–24 years	RYO use only	6–15	7
2	Male	25–35 years	RYO use only	10–20	5
3	Male	25–35 years	RYO use only	7–25	6
4	Female	25–35 years	RYO use only, except one dual user	7–15	6
5	Male	18–24 years	RYO use only	5–25	5
6	Female	18–24 years	RYO use only	6–10	7
7	Male	18–24 years	RYO use only, except one dual user	6–15	7
8	Female	25–35 years	RYO use only	5–9	7

### Materials

**Figure 1.**
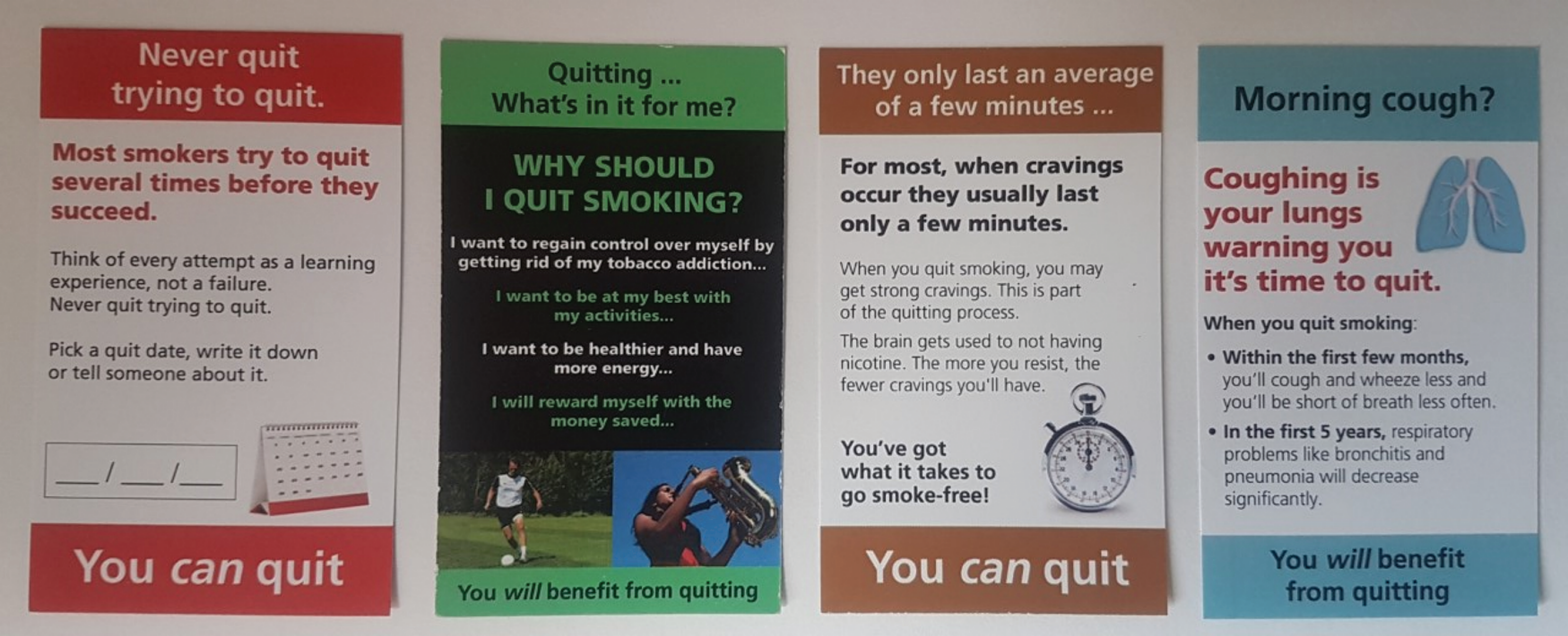
Inserts adapted from those used in Canada.

Four inserts were identical to those used in Canada, except that information at the base of the insert (all mentioned “Health Canada” and two stated that “Nicotine is the drug in tobacco that causes addiction”) was replaced with a self-efficacy (“You can quit”) or response efficacy message (“You will benefit from quitting”), given that efficacy messages on inserts have been found to complement the warnings on packs,^28^ see Figure 1. Two RYO specific inserts were created by the authors, with both intended to help challenge misperceptions about the harm of RYO relative to cigarettes. The first, “Quitting is the most natural choice”, presented “Myths” (that RYO is less addictive, more natural and safer than cigarettes)^20,36,37^ and “Truths” (that levels of tar and nicotine are typically higher in RYO)^23^ about RYO. The second, “Quitting reduces harm”, explained that roll-ups are not less harmful than cigarettes and RYO smoke is toxic, and that quitting reduces harm. This insert featured an image of a roll-up that resembled a tumor, adapted from an image of a tumorous cigarette, to explore the impact of using graphic imagery on an insert, which has not previously been explored.

### Procedure

**Figure 2.**
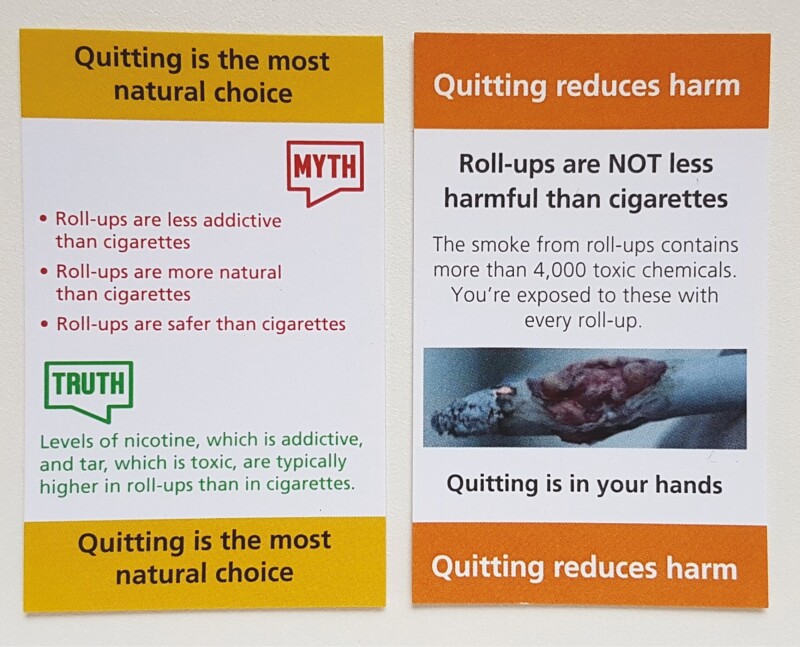
RYO specific inserts.

The market researcher gave eligible participants an information sheet with the study details. Groups took place in a hotel/community center and were moderated, using a semi-structured topic guide, by CM. Participants, who consented to be involved, were informed that their views may differ and when answering they should not be influenced by anyone in the group or the moderator. The discussions were audio recorded, with field notes taken after each group. Focus groups allow participants to interact with stimuli and within each group participants were shown, and allowed to handle, the Canadian inserts (Figure 1) and, following discussion of these, the RYO inserts (Figure 2).

Participants were asked why they use RYO, and given time to discuss this. Each participant was then handed the four Canadian inserts, and told that these were used in cigarette packs in Canada. Groups were asked their views on these inserts, whether they would read them if they appeared in their pack or pouch, what they thought of the messaging, and perceived utility as a cessation aid. Following discussion of these inserts each participant was handed the two RYO inserts and the group asked their perceptions of these and how they compared to the first set. Groups lasted approximately 90 min, with participants receiving an incentive (£30). Ethical approval was obtained from the University of Stirling (GUEP726).

### Analysis

Discussions were transcribed verbatim by a professional transcription agency. The data were then de-identified and imported into NVivo 12 for reflexive thematic analysis. Both authors (RO, CM) familiarized themselves with the data by reading and re-reading transcripts. Preliminary codes were identified by RO and collated into overarching themes and subthemes, which were then refined through detailed re-examination of the data and reflexive discussion with CM. In reporting the results, quotes include participant’s age group (18–24, 25–35) and gender (M or F), with demographic differences mentioned where these exist.

## Results

### Reasons for RYO Use

#### Price

The price of RYO was a key reason for use. While the cost of a pack of RYO is typically higher than for cigarettes, participants explained that RYO lasts significantly longer, thus saving them money. Participants offered multiple reasons for why RYO lasted longer than cigarettes. Aside from the greater amount of tobacco in packs, some participants spoke of rolling shorter or “skinny” roll-ups as required, and others consciously used the fact that roll-ups do not stay continuously lit to smoke them in stages. Participants also explained that the physical act of rolling a cigarette involved a more reflective thought process than simply taking a cigarette from a pack, seen by many as an automatic decision-making process. While price was a driver, most agreed that RYO would remain their preference irrespective of cost.

“*It’s more affordable…*

*Yes.*

*It lasts longer… if I was smoking a packet of fags I’d probably need to buy a pack every two or three days whereas a pack of tobacco lasts two weeks usually*” (FG2, 25-35M).

“*If you get a packet of cigarettes you end up smoking them constantly because they are just there, you don’t have to go out and roll them, you just go ‘oh right okay there you go…’*

*It* [RYO] *just lasts longer*” (FG4, 25-35F).

“*Mine lasts for ages because I just smoke wee skinny ones, they do me. I’m like two weeks on that, maybe even more*” (FG1, 18-24F).

#### Taste

Most preferred the taste of RYO to cigarettes, describing RYO as lighter or smoother and cigarettes as giving a harsh “throat hit”:

“*I prefer rolling because* [with cigarettes] *I feel like dizzy or whatever, I think it’s too strong.*

*I think it’s* [RYO] *easier on your throat if you smoke a lot, its smoother rather than like fags can actually like catch the back of your throat.*

*I just like the taste.*

*Yeah, I just prefer the taste*” (FG8, 25-35F).

“*It feels lighter, like a rollie to me, like smoking a straight it kind of hits the back of your throat, if I go out one night I smoke a twenty deck and then in the morning my throat will be like glass*” (FG1, 18-24F).

One participant spoke of the ability to alter RYO strength according to personal taste:

“*If I want loads* [of tobacco] *and I want it to be really strong then I will just put a roach in it and just no filter*” (FG4, 25-35F).

#### The ritual of rolling

Participants spoke of the pleasure gained from rolling cigarettes. Many described this physical act as a ritual, and gained much satisfaction and pride from refining and perfecting the skill of rolling:

“*When you get one and it’s your first one and it’s perfect, I’m the happiest man alive*” (FG5, 18-24M).

“*When you first start you obviously roll a lot of bad fags, so as you progress on you kind of like that you,*

*You see it as an accomplishment.*

*Yeah. If you roll like a really good fag it’s like…*

*Someone get a photo!*” (FG7, 18-24M).

“*I get made fun of for my rolling skills but… I personally feel like there’s been some progress.*

*I like it because often people will go ‘No, you can’t roll a cigarette with those nails!’ and I’m like watch me! Honestly, watch me!*” (FG8, 25-35F).

Two groups harbored particularly strong RYO smoker identities, and distinguished themselves from cigarette smokers on this basis, with rolling contributing to this identity:

“*I feel like an actual smoker when I’ve got a pouch, see when I see someone with straights I’m like, even though they do smoke it every day, there’s something about it. I think the fact I actually like buy it and I roll it I feel, I feel like I look like I know what I’m doing. I’m a smoker,*

*Yeah, you’ve built it yourself.*

*People that smoke straights are fake*” (FG6, 18-24F).

Some participants spoke about ways in which rolling cigarettes provided psychological relief. The ability to customize each RYO cigarette, according to individual circumstance and context, was also important:

“*It’s quite cathartic… sometimes just rolling the cigarette itself is like a cure for anxiety*” (FG3, 25-35M).

“*There’s a lot more like craft that goes into* [making RYO] *cigarettes…*

*It’s your own wee thing. You’re customising it yourself. It’s yours*” (FG3, 25-35M).

#### Perceptions of harm

All groups discussed the harms associated with RYO compared to cigarettes. RYO was often considered less harmful, and this view was underpinned by the belief that RYO is composed of more natural ingredients and devoid of the chemicals and additives in cigarettes:

“*I thought it* [RYO] *was more better for you… because it’s literally just tobacco*” (FG1, 18-24F).

“*I know it’s* [RYO] *obviously no healthy, but healthier than the straights because I’ve heard that you don’t actually know all the stuff that goes into straights…*

*Baccy* [RYO] *feels a lot more pure if you like. It doesn’t feel like anything’s going into it*” (FG7, 18-24M).

Some participants mentioned the perceived purity of RYO as a motive for switching from cigarettes, eg, “That’s one of the reasons I did it [switched], also the money aspect, but also that, I thought it was just, ‘oh that’s just the baccy, nothing… no other stuff’s in it’” (FG1, 18-24F). A number of females expressed the view that RYO cigarettes are more natural not just because of the tobacco being less adulterated, but as they are hand-made:

“*It feels less processed… even the filter, I prefer smoking from a filter* [I inserted] *myself rather it all being connected…*

*I don’t know what’s in tobacco essentially but because I’m putting that in a cigarette myself and I’m seeing there’s no additives or anything like that… you’ve made it yourself…*

*When I buy it, I take it out of the packet and it’s almost like you can taste the other stuff that’s in there*” (FG8, 25-35F).

#### Inserts

One participant, who had visited Canada, had previously seen some of the Canadian inserts, but they were novel for the remainder of the sample.

#### Salience

Views on the extent to which inserts would capture attention if included in RYO packs or pouches were mixed. Some felt they would be read, at least once, due to their novelty or content, while others thought they would be instantly thrown away; concerns were raised about the environmental impact of discarded inserts.

“*They’re more appealing to look at than fag packets, cigarettes, all the messages on that. So if you wanted to read something about stopping smoking then they’re, it’s a better option I suppose.*

*I think it is good*” (FG1, 18-34F).

“*There will be people who are never going to look at these; the amount of waste is absolutely crazy*” (FG4, 25-35F).

Several participants talked about retaining inserts, and indeed some asked if they could keep the inserts they were given, with one group suggesting that they would be a salient reminder of the benefits of quitting:

“*Like* [inserts inside] *cereal boxes*

*Yeah*

*Collect them all*

*You like collect them and see which ones you get.*

*Can we keep these?*” (FG4, 25-35F).

“*If you were keeping them then eventually you’d have a stack and you’d be like wanting to quit.*

*Yeah, to be honest, it might just be a visual reminder*” (FG7, 18-24M).

One group highlighted the need for rotation to counter habituation: “*If they were changed up more often, there was more variety, then you’d think to look at them*” (FG4, 25-35F).

#### Messaging style

Most valued the messaging used on the Canadian inserts, considered “informative” and “motivational” and often contrasted with on-pack messaging:

“*They wee cards, it is not a case of you’re going to die of cancer, we all know that. It’s more, ‘You can do it’. It’s more encouraging. It’s much more inspirational looking at something like that. I think it resonates a wee bit more with people like ourselves*” (FG3, 25-35M).

“*It would be good to sort of encourage you, a lot of these things are usually quite dismissive, but for some reason I kind of like these.*

*…Yeah, it’s a bit more positive*” (FG5, 18-24M).

Some felt they were already familiar with the information on the inserts, and as such would not pay attention to them:

“*They’re not unhelpful, they’re just kind of telling you something that you already know*” (FG1, 18-24F).

The messaging style of the RYO inserts was, in comparison, viewed unfavorably. Participants questioned the credibility of the information presented on the “myths” and “truths” insert because most believed that RYO is healthier than cigarettes, the statements lacked a source, and they were seen as “scaremongering”:

“*I’ve not just smoked roll ups because that’s just what I’ve fallen into. I’ve smoked them because I’m being told time and time again that although neither of them are great for you, straights have all these other chemicals and toxins and whatever put in that rollies don’t… one little card telling me something else - that’s why I question the credibility*” (FG6, 18-24F).

*“If I read that [myths and truths insert] in an independent study I’d be more likely to believe it.*

*Yeah.*

*But like I would doubt the truth of it simply because it is coming as a warning in a cigarette packet so it’s going to try to be as scary as possible.*

*I’d like you know, ‘find out more at* www.something,something *’ - put that on the bottom of it and I would actually maybe look it up, but because there is nothing,*

*Anybody could have just wrote this*” (FG4, 25-35F).

Some found the RYO insert featuring an image of a tumor difficult to look at, leading one participant to suggest this would “*more likely make me want to stop smoking*” (FG4, 25-35F). However, most stated they were desensitized to graphic warning images, and this was generally viewed as an ineffective attempt to scare RYO smokers into stopping:

“*It’s* [tumour insert] *more about scaring you into quitting rather than being like this is an actual realistic goal you can set yourself,*

*It makes you more dismissive of it*” (FG7, 18-24M).

Several groups suggested ways to improve the RYO inserts, which generally involved replacing messaging perceived as negative with motivational text, and including factual information about the benefits of cessation, eg, “*Something that motivates you to stop smoking*” (FG1, 18-24F). One group suggested extending the Canadian inserts by having a motivational message on one side and “how to quit” messaging on the other:

“*Say they were double-sided… the green one would be really good, like “this* [is] *what you can achieve when you stop smoking”, and then on the back steps of how to go about it.*

*That’s a really good idea actually*

*It’s good to have positivity to get people on board and have it eye-catching, you don’t want to read something negative. I think that, and then the steps to how to go about it on the back, like contact your GP*” (FG8, 25-35F).

#### Perceived impact on own/others’ smoking behavior

Participants generally felt it unlikely that inserts would prompt them to change their smoking behavior. Most participants were disinterested in quitting however, with few indicating that they would like to stop smoking either in the short-term or in the future. Nevertheless, one participant stated that the information on managing nicotine cravings, which was new to him, might have helped during his last quit attempt:

“*If I had known that* [information about cravings being short-lived] *at the time that I decided to stop and I was in my flat and I was like I need a fag, I need to go downstairs and smoke, I’d have probably been like well actually this will pass I think. Give it five minutes and you might be okay*” (FG5, 18-24M).

Other participants thought that inserts might be useful for those contemplating or actively trying to quit, or young people thinking about starting smoking:

“*For people who are trying to quit maybe they will be motivational… the one with the date that we were talking about earlier on, I think that can work as a little goal, even if they don’t succeed on the first attempt. Maybe the next packet they will buy maybe they will make that deadline. Who knows?*

*I think they would be helpful*” (FG8, 25-35F).

“*It’s ideal for young people... they’d probably sit down and read that and go, oh… but for people like us that’s done if for years it’s just not going to make any difference*” (FG2, 25-35M).

One group suggested different impacts of on-pack warnings and inserts, suggesting a role for both:

“*This* [pointing to an on-pack warning] *is maybe to discourage people starting and then the inside* [inserts] *is to encourage people to quit*” (FG3, 25-35M).

## Discussion

Lower price was the key reason for using RYO, consistent with academic^13–16^ and tobacco industry research.^38^ It seems inevitable that this will continue to be the case unless policies aimed at reducing or removing the financial incentive of using or switching to RYO are introduced.^39^ However, many participants indicated that they would continue to use RYO irrespective of price, with other drivers including better taste, the pleasure of rolling, the ability to customize roll-ups, and misperceptions of harm. These findings echo prior research,^17–20,38^ suggesting that reasons for RYO use remain the same in markets with standardized packaging.

Previous research with cigarette smokers in Scotland^31^ and Turkey,^33^ who were shown inserts used in Canada, found that they were thought to capture attention due to their novelty, visibility when opening the pack, and as they would be taken out of packs, with the positive messaging style viewed favorably.^31,33^ The findings were similar among our sample of RYO smokers, although there were mixed perceptions about how salient inserts would be. One explanation for this, and why no participant mentioned visibility when opening the pack, may be that while inserts are prominently displayed in cigarette packs they would likely have reduced visibility in RYO pouches, which dominate the UK market. One solution, as explored by researchers in New Zealand, would be to use the inside of the pouch to communicate health messaging.^40^ This is a viable option, although in previous research cigarette smokers suggested that inserts would extend health messaging as they would be removed from the pack and remain within the household or elsewhere, or be intentionally retained (eg, in their pocket or purse) as a convenient reminder if they were attempting to quit.^31^ In addition, in the United Kingdom and across much of Europe there are already two separate health messages (“Smoking kills – quit now”, and “Tobacco smoke contains more than 70 substances known to cause cancer”) on the inside of pouches, and it would lead to different presentation of this messaging on RYO and cigarette packs. Another option, and potential area for future research, would be to require larger inserts in pouches, which would be feasible given that RYO pouches are much larger than cigarette packs in the United Kingdom (and in the European Union) because the minimum pack weight is 30 g.^41^

Research suggests that the Canadian inserts may help discourage initiation^31,33^ and encourage cessation,^27,28,32^ particularly among those wanting to quit.^31,33^ Our findings were similar, but participants suggested that the inserts would have minimal impact on their own smoking behavior. Compared to cigarette smokers, RYO smokers have a more positive perception of tobacco use,^17^ higher levels of nicotine addiction,^17,18,42^ less confidence that they can quit,^43^ lower intention to quit,^13,42,44^ lower motivation to quit,^45^ and lower incidence of quit attempts.^45^ The RYO smokers in our sample appeared to have very limited interest in quitting, which may help explain the findings with respect to their perceived behavioral impact in response to inserts. Although RYO smokers are less likely than cigarette smokers to make a quit attempt, they are not less likely to succeed in quitting,^17,18,45,46^ and therefore further work on how to promote cessation efforts among this population, whether via inserts or any other means, is warranted.

In the misguided pursuit of a less harmful smoking experience, smokers have switched from unfiltered to filtered cigarettes since the 1950s and turned to low tar products since the 1960s,^47^ with the increasing use of RYO within the last few decades a continuation of this trend. That RYO smokers reported reduced harm as a reason for use was predictable, but the resistance of our sample to the messaging explaining that RYO is not less harmful than cigarettes suggests that this is a strongly held belief. Despite the negative perception of these messages, which directly challenge this belief, we offer no insight into the possible impacts of prolonged exposure to such messaging. Naturalistic research, where RYO smokers have inserts with RYO-specific messages (eg, about harms) placed in their packs or pouches for a period of time, would seem a fruitful area of research. Some participants posited that a source attribution statement would increase the credibility of the information on the RYO inserts. As the guidelines for Article 11 of the Framework Convention on Tobacco Control recommend that beliefs and attitudes among target population subgroups should determine whether the inclusion of such information is likely to be of value,^48^ future research could consider the impact, if any, that the inclusion of a source attribution statement makes when presenting information about RYO harms. Further testing of other aspects of insert design, such as message style (eg, relative vs absolute) and framing (eg, positive vs negative framing), and inclusion of efficacy statements, would also be of value. However, with respect to the graphic imagery used on one of the RYO inserts, as this was viewed negatively and thought to reduce engagement we do not consider this a fruitful area of future research.

In terms of limitations, focus groups are not generalizable. While we focused on young adults as they are most likely to be RYO smokers, we are unable to provide any insight into how middle- and older-aged RYO smokers, and those below the age of 18, may respond to inserts. The novelty of the inserts may have also influenced responses, as new stimuli can attract favorable or unfavorable attention and distort findings.^49^ In addition, for practical reasons the RYO inserts were not professionally designed or pre-tested with the target population,^48^ which may have influenced participants’ responses, and participants were only shown RYO inserts about harm. The presentation order of questions and messages could have biased responses, for instance by affirming values or reasons for use prior to presenting risk information.

RYO is an increasingly popular product in the United Kingdom and elsewhere, with the global RYO market predicted to grow faster than the total tobacco market from 2021 to 2028,^50^ driven by increased use among females and depressed incomes.^51,52^ As more governments legislate for, or are moving toward, pack inserts, work is needed to understand how best to incorporate these within RYO pouches, what RYO specific inserts appear to work best, and the potential impacts of such inserts among this population.

## Supplementary Material

A Contributorship Form detailing each author’s specific involvement with this content, as well as any supplementary data, are available online at https://academic.oup.com/ntr.

ntac184_suppl_Supplementary_Taxonomy_Form

## Data Availability

The data underlying this article will be shared on reasonable request to the corresponding author upon the publication of all planned research from the project.
